# Levels and trends in cardiovascular risk factors and drug treatment in 4837 elderly Dutch myocardial infarction patients between 2002 and 2006

**DOI:** 10.1007/s12471-012-0248-z

**Published:** 2012-02-08

**Authors:** S. S. Soedamah-Muthu, J. M. Geleijnse, E. J. Giltay, J. de Goede, L. M. Oude Griep, E. Waterham, A. M. Teitsma-Jansen, B. J. M. Mulder, M.-J. de Boer, J. W. Deckers, P. L. Zock, D. Kromhout, for the Alpha Omega Trial Group

**Affiliations:** 1 Division of Human Nutrition, Wageningen University, PO Box 8129, 6700 EV Wageningen, the Netherlands; 2 Department of Psychiatry, Leiden University Medical Center, Leiden, the Netherlands; 3 Department of Cardiology, Academic Medical Center, Amsterdam, the Netherlands; 4 University Medical Center St Radboud Nijmegen, Nijmegen, the Netherlands; 5 Department of Cardiology, Erasmus Medical Center, Rotterdam, the Netherlands; 6 Unilever Research and Development, Vlaardingen, the Netherlands

**Keywords:** Trends, Coronary heart disease patients, Myocardial infarction, Risk factors, Medication

## Abstract

**Background:**

It is important to gain insight into opportunities for secondary prevention of cardiovascular disease. Our aim was to investigate levels and trends in cardiovascular risk factors and drug treatment in Dutch post-myocardial infarction (MI) patients between 2002 and 2006 and to make comparisons with the EUROASPIRE surveys (1999–2007).

**Methods:**

We analysed data from 4837 post-MI patients (aged 69 years, 78% men) from 32 Dutch hospitals, using baseline cross-sectional data from the Alpha Omega Trial.

**Results:**

Between 2002 and 2006, significant declines were found in the prevalence of smoking (23% to 16%, *p* < 0.001), hypercholesterolaemia (≥5 mmol/l; 54% to 27%, *p* < 0.0001) and hypertension (≥140/90 mmHg; 58% to 48%, *p* < 0.001). The prevalence of antithrombotic drugs was high (97%). The prevalence of lipid-modifying drugs and antihypertensives was high, and increased (74% to 90%, *p* < 0.0001 and 82% to 93%, *p* < 0.001, respectively). The prevalence of obesity (27%) was high in 2002 and decreased to 24% in 2006, albeit not significantly. Diabetes prevalence was high and increased between 2002 and 2006 (18% to 22%, *p* = 0.02). In comparison with EUROASPIRE patients, who were on average 8–10 years younger, our study in 2006 included patients with lower levels of obesity, hypertension, hypercholesterolaemia, diabetes and lower use of antiplatelets and β-blockers, but similar levels of lipid-modifying drugs.

**Conclusions:**

This study showed that older Dutch post-MI patients were adequately treated with drugs, and that risk factors reached lower levels than in the younger EUROASPIRE patients. However, there is room for improvement in diet and lifestyle, given the high prevalence of smoking, obesity, and diabetes.

**Electronic supplementary material:**

The online version of this article (doi:10.1007/s12471-012-0248-z) contains supplementary material, which is available to authorized users.

## Introduction

Cardiovascular diseases (CVD) claim over 4 million lives in Europe every year [1, 2]. In the Netherlands, CVD caused 346,000 hospitalisations and accounted for 30% of total mortality in 2008 [3]. Although mortality due to CVD is declining in most European countries, the number of CVD patients is still increasing [3–5]. The decline in mortality could be due to improved prognosis and better management of CVD [3, 4]. The increasing prevalence of CVD is due to ageing populations and improved survival of patients with CVD [3, 4]. It is unknown how trends in risk factors and drug treatment have recently evolved over time in the Netherlands, especially in older patients with coronary heart disease (CHD) (mean age 69 years), and how these compare with other European countries.

The largest study in Europe examining trends in risk factors and drug treatment in CHD patients is the EUROASPIRE study. Independent cross-sectional surveys were carried out in eight European countries in 2975 CHD patients (1999–2000, mean age 59 years) and in 2392 CHD patients (2006–2007, mean age 61 years) [6]. In 2007, the prevalence for smoking was 18%, for obesity 38%, for elevated blood pressure (BP ≥140/90 mmHg) 61%, for hypercholesterolaemia 46% and for self-reported diabetes mellitus 28%. Compared with 1999, obesity and diabetes had increased (both by 6%) and hypercholesterolaemia had decreased (by 32%), whereas smoking and elevated BP remained stable. On the basis of EUROASPIRE it was recommended that more effective lifestyle interventions were needed for CHD patients, i.e. smoking cessation, weight loss and prevention of diabetes [6]. EUROASPIRE included only 387 Dutch CHD patients and conclusions specific to this population could therefore not be drawn [6].

From a clinical perspective it is important to gain insight into opportunities for secondary prevention of CVD. Therefore, we assessed time trends and levels of CVD risk factors and drug treatment in 4837 post-myocardial infarction (MI) patients in the Netherlands between 2002 and 2006. A second aim was to compare these time trends and levels with those in EUROASPIRE in the period 1999–2007.

## Methods

### Study design and population

We used baseline cross-sectional data of the Alpha Omega Trial (ClinicalTrials.gov identifier: NCT00127452, see website http://www.alphaomegatrial.com), a multi-centre trial of n-3 fatty acids and CVD endpoints [7, 8]. The study involved 4837 Dutch men and women aged 60–80 years with a documented history of MI who were recruited from 32 Dutch hospitals between April 2002 and December 2006. Written informed consent was obtained from each subject. The study was conducted in accordance with the Helsinki Declaration and approved by the central Medical Ethics Committee South-West Holland and by the local medical ethics committees of participating hospitals (see online supplementary material under participating cardiology centres).

### Measurements

Patients were physically examined by trained research nurses in the hospital or at home, according to a standard protocol [7]. Information was obtained on current health status, medical history, lifestyle and drug treatment.

Weight and height were measured once with patients wearing light clothes and no shoes. Body mass index (BMI) was calculated as weight (kg)/height^2^ (m). Obesity was defined as BMI ≥30.0 kg/m^2^, and morbid obesity as BMI ≥40.0 kg/m^2^. Waist circumference was measured at the midpoint between the bottom rib and the top of the hipbone. Central obesity was defined as a waist circumference of ≥88 cm in women or ≥102 cm in men [9]. Systolic and diastolic BP (1st and 5th Korotkoff sounds, respectively) were measured twice at the left upper arm after a 10-min seated rest with an automated device (Omron HEM-711, Omron Healthcare Europe B.V., Hoofddorp, NL). Values were averaged. Elevated BP levels (or hypertension, as in EUROASPIRE [6]) were defined as systolic BP ≥140 mmHg and/or diastolic BP ≥90 mmHg.

Casual venous blood samples were taken for the assessment of serum lipids and plasma glucose as described in detail previously [7]. Serum total cholesterol, high-density lipoprotein cholesterol (HDL-c), triglycerides and glucose were analysed by standard kits using an autoanalyser (Hitachi 912, Roche Diagnostics, Basel, Switzerland). Low-density lipoprotein cholesterol (LDL-c) was calculated using the Friedewald formula [10]. Elevated cholesterol (or hypercholesterolaemia, as in EUROASPIRE [6]) was defined as total serum cholesterol ≥5.0 mmol/l (193 mg/dl).

Patients filled out questionnaires on smoking status (current, former or never), educational level, drug treatment, dietary advice prescribed by the physician, and history of CVD, hypercholesterolaemia, hypertension and diabetes mellitus. All of these were checked and completed, if needed, by research nurses. Diabetes was defined as self-reported physician diagnosis, drug treatment or glucose ≥7.0 mmol/l (126 mg/dl) for fasting and ≥11.1 mmol/l (200 mg/dl) for non-fasting patients. Self-reported diabetes was presented to compare with EUROASPIRE. The majority of the diabetes cases (73%) were based on a combination of these criteria and the remaining 27% on either self-reporting, or glucose values or drug treatment. Self-reported drug treatment of the participants was coded by a pharmaco-epidemiologist according to the Anatomical Therapeutic Chemical Classification System (ATC) [11]. ATC codes were C02, C03, C07, C08, and C09 for antihypertensive drugs, C10 for lipid-modifying drug treatment (with C10AA coding for statins and C10B coding for combinations of statins with other lipid-modifying drugs), A10 for antidiabetic drug treatment and B01 for antithrombotic drug treatment.

### Statistical analysis

To assess trends over time in CVD risk factors and drug treatment use, we categorised patients according to enrolment year. General linear models (GLM) were used to estimate mean changes for continuous variables and percentage changes for dichotomous variables between 2002 and 2006, all adjusted for year, age and gender by entering these as covariates in the GLM models. Tukey’s method was used to estimate percentage or mean differences from 2006 to 2002 [12].

We compared our data with those from the EUROASPIRE II (1999/2000) and III surveys (2006/2007) [6]. In EUROASPIRE, changes over time in risk factors and drug treatment had been adjusted using the same GLM and Tukey’s method with survey, age and diagnostic category as the main covariates [6].

P for trend was calculated with age- and gender-adjusted linear (for continuous variables) or logistic (for binary variables) regression models with year as the independent variable. We also analysed CVD risk factors and drug treatment by gender. *P*-values for interaction were calculated from the linear regression models (term gender*year) and *p*-values <0.2 were considered indicative for interaction.

For all other analyses, two-sided *p*-values <0.01 indicated statistical significance. SAS version 9.1 (SAS Institute, Cary, North Carolina, USA) was used for all statistical analyses.

## Results

Patients entering the Alpha Omega Trial were on average 69.0 [SD 5.6] years and 78% were male. The prevalence of central obesity (based on waist circumference) was 60%, the prevalence of obesity (based on BMI) was 24% and for morbid obesity it was 0.8%. Most patients were included in 2005 and 2006 (60%). Characteristics of the study population by the year of enrolment are described in Table 1. Time since MI at enrolment decreased from 5.6 [SD 3.9] years in 2002 to 3.7 [SD 3.0] years in 2006.

**Table 1:** Study characteristics and CVD risk factors of 4837 post-MI patients included in the Alpha Omega Trial between 2002 and 2006 by year of enrolment

	2002	2003	2004	2005	2006	*P* for trend
N	522	759	674	1398	1484	
Age (years)	69.4 (5.5)	69.3 (5.6)	69.3 (5.6)	68.6 (5.2)	69.0 (5.8)	
Men%(n)	76 (395)	76 (577)	79 (532)	80 (1124)	78 (1155)	
High education% (n)	10 (50)	12 (93)	11 (73)	13 (185)	13 (196)	
Time since MI (years)	5.6 (3.9)	4.8 (3.1)	4.1 (2.7)	4.2 (3.1)	3.7 (3.0)	
Prescribed diet% (n)	12 (61)	14 (102)	11 (71)	10 (145)	13 (186)	
Cardiovascular risk factors						
Current smoking% (n)	23 (121)	17 (131)	18 (121)	15 (208)	16 (231)	<0.001^a^
BMI (kg/m^2^)	27.9 (3.9)	28.1 (3.9)	27.8 (3.9)	27.6 (3.7)	27.7 (3.9)	0.041
Obesity%(n)	27 (141)	26 (198)	23 (153)	23 (326)	24 (352)	0.08
Waist circumference (cm)	101.4 (10.5)	101.3 (10.5)	101.8 (10.3)	102.0 (10.3)	102.5 (10.8)	0.0096^a^
Central obesity% (n)	59 (308)	58 (442)	60 (407)	59 (828)	60 (893)	0.28
Glucose (mmol/l)	6.07 (2.06)	6.16 (1.99)	6.29 (2.27)	5.98 (2.07)	6.47 (2.08)	0.001^a^
Diabetes patients% (n)	18 (94)	21 (158)	24 (165)	19 (267)	22 (330)	0.19
Self-reported stroke% (n)	7 (36)	9 (67)	7 (46)	6 (83)	8 (113)	0.86
Total cholesterol (mmol/l)	5.18 (1.05)	5.09 (0.92)	4.81 (0.92)	4.54 (0.91)	4.51 (0.93)	<0.001^a^
Elevated cholesterol% (n)	54 (275)	50 (372)	35 (233)	27 (358)	27 (389)	<0.001^a^
LDL-c (mmol/l)	3.03 (0.91)	2.91 (0.80)	2.72 (0.78)	2.47 (0.77)	2.31 (0.77)	<0.001^a^
HDL-c (mmol/l)	1.27 (0.34)	1.30 (0.35)	1.23 (0.33)	1.24 (0.33)	1.36 (0.34)	<0.001^a^
Triglycerides (mmol/l)#	1.69 (1.28, 2.37)	1.70 (1.27, 2.38)	1.67 (1.21, 2.31)	1.62 (1.20, 2.28)	1.61 (1.18, 2.31)	0.002^a^
Systolic BP (mmHg)	143.8 (21.6)	145.7 (21.9)	142.8 (21.1)	140.7 (21.0)	139.1 (22.0)	<0.001^a^
Diastolic BP (mmHg)	81.8 (11.3)	82.2 (11.2)	80.8 (10.6)	80.3 (10.8)	78.1 (11.5)	<0.001^a^
Elevated BP% (n)	58 (303)	62 (474)	55 (372)	50 (699)	48 (710)	<0.001^a^

Values represent mean (SD) # median (IQR), or percentage (n). *P* for trend is calculated with age- and gender-adjusted linear (for continuous variables) or logistic (for binary variables) regression models with time in years as the independent variable

*LDL-c* low density lipoprotein cholesterol

*HDL-c* high density lipoprotein cholesterol

*BP* blood pressure

*IQR* interquartile range, percentiles p25–p75

*SD* standard deviation

*BMI* body mass index

To convert the values for glucose to milligrams per decilitre, divide by 0.05551

To convert the values for cholesterol to milligrams per decilitre, divide by 0.02586

To convert the values for triglycerides to milligrams per decilitre, divide by 0.01129

Diabetes was defined on basis of self-reported physician diagnosis, the use of antidiabetic drugs, or newly diagnosed diabetes (fasting glucose ≥7.0 mmol/l or non-fasting ≥11.1 mmol/l)

Obesity was defined as BMI ≥30 kg/m^2^

Central obesity was defined as a waist circumference of ≥88 cm in women or ≥102 cm in men

Elevated cholesterol was defined as serum total cholesterol ≥5.0 mmol/l

Elevated BP was defined as systolic/diastolic BP ≥140/90 mmHg

^a^Statistically significant trend over time if *p* < 0.01

### CVD risk factors

The Alpha Omega Trial showed significant declines (*p* < 0.001) in lipid levels between 2002 and 2006 (Table 1), i.e. −0.67 mmol/l (26 mg/dl) in serum total cholesterol, −0.72 mmol/l (28 mg/dl) in LDL-c and systolic and diastolic BP levels (−4.5 and −3.9 mmHg, respectively, p < 0.001). Elevated cholesterol (54% to 27%, *p* < 0.0001), elevated BP levels (58% to 48%, *p* < 0.0001), and current smoking (23% to 16%, *p* = 0.0003) also decreased. The prevalence of obesity (27%) and central obesity (59%) started at high levels in 2002, but decreased slightly or remained constant, respectively, over the total time period. Approximately 12% were receiving dietary advice from the physician. The prevalence of diabetes was high (18%) and increased to 22% (*p* = 0.02).

### Drug treatment

Drug treatment started at high levels in 2002 (Table 2). Antithrombotic drugs were prescribed to 97% of the patients in 2002 and this remained constant throughout the years. Increases were found for lipid-modifying drugs (74% to 90%, *p* < 0.0001) and antihypertensives (82% to 93%, *p* < 0.0001). Of the lipid-modifying drugs, 98% consisted of statins in the Alpha Omega Trial. Among the antihypertensive drugs, the largest increase between 2002 and 2006 was found for β-blockers (+21%) and angiotensin II receptor blockers (+9%). Antidiabetic treatment started at 12% in 2002 and increased to 16% in 2006. The increase was mainly due to biguanides (metformin) (+4.8%) and insulin (+3.6%).

**Table 2:** Prescribed cardiovascular drugs of 4837 post-MI patients included in the Alpha Omega Trial between 2002 and 2006 by year of enrolment

	2002	2003	2004	2005	2006	*P* for trend
Number of patients	522	759	674	1398	1484	
Antiplatelet	81 (424)	82 (621)	84 (564)	85 (1186)	85 (1255)	0.035
Antithrombotics	97 (507)	96 (728)	98 (661)	98 (1369)	98 (1453)	0.035
β-blockers	54 (281)	62 (472)	71 (481)	71 (995)	75 (1114)	<0.001^a^
ACE inhibitors	40 (211)	40 (305)	39 (263)	47 (656)	43 (631)	0.043
ARBs	9 (48)	13 (102)	15 (101)	14 (198)	18 (265)	<0.001^a^
ACEIs and ARBs	49 (257)	53 (403)	53 (354)	60 (834)	59 (878)	<0.001^a^
CCBs	17 (89)	19 (143)	26 (172)	20 (283)	19 (280)	0.72
Diuretics	30 (155)	24 (182)	24 (160)	21 (299)	26 (388)	0.58
All antihypertensive drugs	82 (430)	85 (647)	90 (607)	91 (1271)	93 (1385)	<0.001^a^
All lipid-modifying drugs	74 (384)	81 (616)	87 (586)	89 (1244)	90 (1331)	<0.001^a^
Insulin	2 (13)	5 (36)	7 (44)	7 (91)	6 (89)	0.004^a^
Biguanides	5 (24)	8 (62)	9 (60)	8 (118)	9 (139)	0.005^a^
Sulphonamides	9 (49)	8 (64)	9 (58)	7 (91)	7 (99)	0.016
All antidiabetic drugs	12 (61)	15 (116)	18 (121)	15 (206)	16 (236)	0.11

*ACEI* angiotensin-converting enzyme inhibitor; *ARB* angiotensin II receptor blocker

^a^Statistically significant trend over time, *p*<0.01

*P* for trend is calculated with age- and gender adjusted logistic (for binary variables) regression models with year as the independent variable

### Differences between men and women

Men (*n* = 3783) and women (*n* = 1054) had similar levels of risk factors and drug treatment in 2002, except for elevated cholesterol which was higher in women than in men (63% vs. 50%, *p* = 0.01). The number of patients with elevated cholesterol was halved in 2006 in both men and women, but remained higher in women than in men (33% vs. 25%, *p* = 0.008), despite similar prescription rates for lipid-modifying drugs in men and women (in 2006; 90% and 87% respectively). Systolic BP levels in 2006 were significantly higher in women (mean 140.6 [SD 23.6] mmHg) than in men (mean 138.7 [SD 21.5] mmHg, *p* = 0.04), but antihypertensive prescription rates in 2006 were similar (93%).

Trends in risk factors and medication between 2002 and 2006 were generally similar in men and women.

### Comparison with EUROASPIRE

EUROASPIRE patients were 8–10 years younger (59 and 61 years in survey II and III, respectively) than our patients (69 years), but they had a similar gender distribution (75% and 77% men, respectively, in the EUROASPIRE surveys) compared with 78% in the Alpha Omega Trial (Table 3). Besides post-MI patients, the EUROASPIRE study population also consisted of patients who underwent PCI, and patients who had angina pectoris or acute coronary syndrome [6]. We found similar initial risk factor levels comparing the Alpha Omega Trial (2002) to EUROASPIRE II (1999–2000) data, except for a lower prevalence of elevated cholesterol, self-reported diabetes and β-blocker use. We also found a higher initial prescription rate for lipid-modifying drugs in the Alpha Omega Trial (2002) compared with EUROASPIRE II (1999–2000; 74% vs. 63%).

**Table 3:** CVD risk factor and drug prescription levels and changes over time in post-MI patients, comparison of Alpha Omega Trial (AOT) and EUROASPIRE using similar definitions

	AOT	AOT	AOT	EUROASPIRE	EUROASPIRE	EUROASPIRE
	2002	2006	Change from 2002 to 2006	1999–2000	2006–2007	Change from 1999 to 2007
	*N* = 522	*N* = 1484	*N* = 1484	*N* = 2975	*N* = 2392	*N* = 2392
Age	69.4 (5.5)	69.0 (5.8)		59.4 (8.5)	60.9 (7.6)	
Male gender	76% (395/522)	78% (1155/1484)		75% (2223/2975)	77% (1831/2392)	
Current smoking	23% (121/522)	16% (231/1483)	−7.9% (−12.4, −3.4)^a^	21% (631/2971)	18% (434/2381)	−1.6% (−5.3, 2.2)
Obesity	27% (141/520)	24% (352/1481)	−3.3% (−8.4, 1.9)	33% (967/2963)	38% (902/2376)	5.5% (−0.1, 11.1)
Elevated BP	58% (303/521)	48% (710/1482)	−10.0% (−16.0, −4.0)^a^	58% (1730/2969)	61% (1452/2385)	3.4% (−3.2, 9.9)
Elevated cholesterol	54% (275/514)	27% (389/1438)	−26.3% (−32.0, −20.6)^a^	77% (2122/2766)	46% (1049/2273)	−32.4% (−39.0, −25.9)^a^
Self-reported diabetes mellitus	14% (74/519)	19% (274/1466)	4.7% (0.07, 9.3)^a^	20% (598/2970)	28% (664/2371)	6.4% (1.4, 11.4)^a^
Antiplatelet drugs%	81% (424/522)	85% (1255/1484)	3.1% (−1.4, 7.6)	84% (2486/2973)	93% (2214/2376)	7.1% (4.0, 10.2)^a^
Lipid-modifying drugs%	74% (384/522)	90% (1331/1484)	15.8% (11.7, 20.0)^a^	63% (1864/2973)	89% (2110/2376)	23.7% (19.2, 28.1)^a^
β-blockers%	54% (281/522)	75% (1114/1484)	21.2% (15.6, 26.8)^a^	69% (2052/2973)	86% (2031/2376)	15.1% (8.5, 21.6)^a^
Antihypertensives%	82% (430/522)	93% (1385/1484)	11.1% (7.4, 14.8)^a^	91% (2694/2973)	97% (2301/2376)	5.5% (2.4, 8.5)^a^

EUROASPIRE data were adapted from Lancet 2009: 373:929 by K. Kotseva [6]

^a^Statistically significant if confidence interval does not include zero

*BP* blood pressure

Changes were indicated as mean or percentage changes (95% CI) from age- and gender adjusted linear regression models

Achieved levels and trends were compared between 2002 and 2006 in the Alpha Omega Trial versus a longer period (1999–2007) in EUROASPIRE. In the Alpha Omega Trial, we observed significant age- and gender-adjusted declines for smoking, elevated BP and elevated cholesterol and significant increases in the prevalence of self-reported diabetes, lipid-modifying drugs, antihypertensives and β-blockers. Obesity tended to decline in our study, although not significantly. In EUROASPIRE, non-significant declines were found for smoking, and non-significant increases in obesity and hypertension rates. Significant declines were demonstrated in EUROASPIRE for hypercholesterolaemia and significant increases in the prevalence of self-reported diabetes, antiplatelets, lipid-modifying drugs, antihypertensives and β-blockers. Achieved levels by 2006–2007 were similar in both studies for smoking and lipid-modifying drugs. The achieved prevalence of obesity, diabetes, elevated BP and elevated cholesterol was lower in our study than in EUROASPIRE. The achieved prescription rates of antiplatelets and β-blockers and to a lesser extent antihypertensives were lower in our study than in EUROASPIRE.

## Discussion

In this large Dutch population of post-MI patients, we found high drug prescription levels, in particular for antithrombotics, lipid-modifying drugs and antihypertensives. Over a 4-year period (2002–2006), increases in the use of lipid-modifying drugs and antihypertensives corresponded with declines in serum cholesterol (LDL) and BP levels. Smoking prevalence decreased over time, whereas (central) obesity remained relatively stable and the prevalence of diabetes increased. In comparison with the on average 10 year younger EUROASPIRE patients, lower levels of obesity, elevated BP, elevated cholesterol and diabetes, and lower prescription rates of antiplatelets and β-blockers, were observed in the Alpha Omega Trial in 2006. The prevalence of smoking and lipid-modifying drugs, however, were similar in the two studies [6].

A strength of the present study is the large sample size in stable post-MI patients in the Netherlands. A limitation is that our population is not a random sample of all MI patients in the Netherlands, because it includes only patients who were willing to participate in a 40-month trial [13]. Data on drug prescriptions and smoking behaviour have the well-known limitations of self-reporting. To limit this potential bias we used a standard protocol and calibrated devices for risk factor measurements and all questionnaires were checked by trained research nurses and prescribed medication was classified by an independent pharmaco-epidemiologist according to the WHO ATC coding system.

We compared the baseline data of the Alpha Omega Trial with those of EUROASPIRE, the largest study in Europe in CHD patients, using similar definitions. However this comparison needs to be interpreted carefully, because EUROASPIRE patients differ from those in the Alpha Omega Trial in terms of age, duration of follow-up and type of CHD patients (not only MI patients). A limitation to our study is that we used baseline cross-sectional data from the Alpha Omega Trial and not longitudinal data on the same patients. Advantages are that these can be viewed as cross-sectional surveys as in EUROASPIRE and eligible for estimating trends over a reasonable time period.

### Hypercholesterolaemia

Between 2002 and 2006, we showed a decreasing trend in serum cholesterol (LDL) level and an increase in the use of lipid-modifying drugs. In 2006, the prevalence of hypercholesterolaemia was lower than in EUROASPIRE, possibly due to a higher use of lipid-modifying drugs, for which the effect in secondary prevention of CHD in elderly patients (aged 65–80 years) has been established [14].

The Netherlands (33%) together with Finland (28%) had the lowest prevalence of hypercholesterolaemia in EUROASPIRE in 2006–2007. In descending order the prevalence was 57% for Hungary, 49% for Germany and Italy, 47% for Czech Republic, 42% for Slovenia and 41% for France [6].

These results together with our steep decreases in serum cholesterol levels and increases in lipid-modifying drug use suggest that serum cholesterol levels are well controlled in Dutch patients.

### Hypertension

We showed a decreasing trend in average systolic and diastolic BP (−4 mmHg, *p* for trend <0.001). The 10% significant increase in antihypertensive drugs coincided with a significant decline in elevated BP levels. Starting at a similar rate of elevated BP as in EUROASPIRE, the prevalence of elevated BP reached lower levels in the Alpha Omega Trial than in EUROASPIRE (48% vs. 61%). The EUROASPIRE surveys showed that the prevalence of hypertension remained stable from 1999 to 2007, despite increases in the use of all antihypertensives (except for calcium antagonists) [6]. Country-specific analyses in EUROASPIRE showed that in some countries (Czech Republic, Finland, Hungary, Italy, the Netherlands) the prevalence of hypertension increased over time whereas in other countries (i.e. France, Germany, and Slovenia) the rates decreased [6].

At a drug-specific level, the use of β-blockers increased in our cohort (21%), but was still lower than in EUROASPIRE (75% vs. 85%) in 2006 [6]. In EUROASPIRE, increases in the use of ACE inhibitors and angiotensin-II receptor blockers and diuretics and a decrease in the use of calcium channel blockers were reported. The trends for these drugs were comparable with those in our study.

In summary, between 2002 and 2006 antihypertensive drug use increased strongly in the Netherlands, but the use of β-blockers was still lower than in other European countries.

### Smoking

Smoking rates declined more strongly (from 23% to 16%) over a shorter time period in the Alpha Omega Trial than in EUROASPIRE (from 21% to 18%). At a country-specific level, a similar prevalence of smoking was reported for the Dutch patients in EUROASPIRE III (15%). The prevalence of smoking in the Netherlands was lower compared with other European countries, with the highest prevalence in France in 2007, i.e. 25% [6]. In spite of the relatively low percentage of smokers among post-MI patients in the Netherlands, there is still substantial health gain to be obtained by further refraining from smoking.

### Obesity and diabetes

The prevalence of obesity (27%) was high in 2002 in our study and did not change over the years. In EUROASPIRE, obesity rates started higher (33% in 1999) and increased further to 38% in 2007 [6]. The highest obesity rates were reported in 2006–2007 in Hungary (49%) and Germany (43%), with the Netherlands (27%) and Finland (26%) having the lowest prevalence [6]. Using waist circumference cut-off points, we found a high prevalence of central obesity in 2002 (59%), which remained stable over the years.

The prevalence of self-reported diabetes did increase over the study period from 14% to 19%. At a country-specific level, the Netherlands had the lowest reported prevalence of diabetes in all EUROASPIRE survey years (13% in 1999 and 21% in 2007), whereas Hungary had the highest prevalence (45%) in 2007 [6].

These results suggest that even in the Netherlands with one of the lowest prevalences of obesity and diabetes, central obesity is still highly prevalent. As obesity and physical inactivity are the main risk factors for diabetes [15], a healthy diet and lifestyle is needed to reduce the high absolute mortality risk of obese and diabetic post-MI patients.

### Importance of healthy diet and lifestyle in secondary prevention of CVD

Lifestyle and dietary recommendations for secondary prevention of CVD are: to stop smoking, to increase physical activity and to eat healthily [16]. A healthy diet includes a wide variety of foods and recommendations to reduce saturated and trans fat and salt intake and encourage fruits and vegetables, wholegrain cereals and bread, (oily) fish, lean meat, and low-fat dairy products [16, 17]. A review on the effect of lifestyle measures on all-cause mortality rates showed that increased physical activity reduced all-cause mortality by 25% in CHD patients [17]. For dietary interventions, a recent meta-analysis demonstrated that each 5% increment in polyunsaturated fatty acid consumption reduced CHD risk by 10% in secondary prevention populations [18]. A meta-analysis of trials in CHD patients adding 1–2 grams of n-3 fatty acids per day reduced CHD mortality by 20% [19]. This emphasises the importance of lifestyle and dietary interventions in post-MI patients to lower their absolute risk even further than with drug treatment alone.

### Conclusions

This large population of post-MI patients in the Netherlands is treated well pharmacologically. Moreover, both CVD risk factors and drug prescription rates showed improvements over a 4-year period. Until now, lifestyle interventions in post-MI patients have received little attention as evident from the high proportion of patients with obesity and diabetes and the steep increase in diabetes prevalence. In spite of the relatively favourable risk factor and drug use levels in post-MI patients in the Netherlands compared with other European countries, further improvements of lifestyle and dietary habits will improve the prognosis of these patients.

## Electronic supplementary material

Below is the link to the electronic supplementary material.

ESM 1 (DOC 31 kb)
