# Supplementary material for: Levels and trends in cardiovascular risk factors and drug treatment in 4837 elderly Dutch myocardial infarction patients between 2002 and 2006
Source: Neth Heart J. 2012 Feb 8;20(3):102–9. doi: 10.1007/s12471-012-0248-z (PMC3286508; doi:10.1007/s12471-012-0248-z)
Supplement: Supplementary file 1 — (DOC 31 kb) [file 12471_2012_248_MOESM1_ESM.doc]

**Online Supplementary Material. List of collaborators**

**Alpha Omega Trial Group**

*Executive Committee*

D. Kromhout, Principal Investigator, Division of Human Nutrition, Wageningen University

E.G. Schouten (from 2002-2005), Co-Principal Investigator, Division of Human Nutrition, Wageningen University

J.M. Geleijnse, Trial Coordinator, Division of Human Nutrition, Wageningen University

E.J. Giltay, Study Physician, Department of Psychiatry, Leiden University Medical Center, Leiden

J. de Goede, Trial Assistant, Division of Human Nutrition, Wageningen University

L.M. Oude Griep, Data Quality Monitor, Division of Human Nutrition, Wageningen University

A.M. Teitsma-Jansen, Logistics Manager, Division of Human Nutrition, Wageningen University

E. Waterham, Data Manager, Division of Human Nutrition, Wageningen University

*Steering Committee*Voting members:

B.J.M. Mulder (chair), Academic Medical Center, Amsterdam

J.W. Deckers, Erasmus Medical Center, Rotterdam

M.B. Katan, VU University, Institute for Health Sciences, Amsterdam

P.L. Zock, Division of Human Nutrition, Wageningen University (until January 2004)

Observers:

M.J. de Boer, Isala Clinics, Zwolle

H. de Leeuw, Netherlands Heart Foundation, The Hague

E.G. Schouten, Food and Consumer Product Safety Authority, The Hague (since January 2005)

P.L. Zock, Unilever R&D, Vlaardingen (since January 2004)

*Data and Safety Monitoring Board*

E. Boersma (chair), Erasmus Medical Center, Rotterdam

J.W. Jukema, Leiden University Medical Center, Leiden

J.J. van Binsbergen, Radboud University Medical Center, Nijmegen

*Endpoint Adjudication Committee*

D.A.M. van der Kuip (chair), Rotterdam

K. Thomas, Diaconessenhuis, Meppel

M. Rivero-Ayerza (until January 2009), Erasmus Medical Center, Rotterdam

A.M. Vollaard (since January 2009), Academic Medical Center, Amsterdam

*Independent physician*

C.J. Fieren, Wageningen

*Participating cardiology centers*

Alysis Zorggroep, Kliniek Velp, Velp: L.H.J. van Kempen

BovenIJ Ziekenhuis, Amsterdam: A. Bakx

Bronovo Ziekenhuis, The Hague: M.I. Sedney

Canisius Wilhelmina Ziekenhuis, Nijmegen: D.P. Hertzberger

Catharina-ziekenhuis, Eindhoven: H.R. Michels

Diaconessenhuis, Leiden: A.A. de Rotte, R.P. van Rugge

Erasmus Medisch Centrum, Rotterdam: A. Klootwijk

Flevoziekenhuis, Almere: J.A. Verheul

Gelre Ziekenhuizen, Apeldoorn: D.M. Nicastia

Haga Ziekenhuis, location Leyweg, The Hague: R. Robles de Medina

Haga Ziekenhuis, location Sportlaan, The Hague: M. van Rossem

Havenziekenhuis, Rotterdam: C.M. Leenders

Isala Klinieken, location De Weezenlanden, Zwolle: M.J. de Boer

't Lange Land Ziekenhuis, Zoetermeer: P. van der Meer

Lievensberg Ziekenhuis, Bergen op Zoom: S.C. Uppal, J.G. Blok

Máxima Medisch Centrum, Veldhoven: R.F. Visser

Meander Medisch Centrum, Amersfoort: A. Mosterd

Medisch Centrum Alkmaar, Alkmaar: V.A.W.M. Umans, C.L.A. Reichert

Medisch Spectrum Twente, Enschede: J.W. Louwerenburg

Oosterscheldeziekenhuis, Goes: A.H. Liem

Rijnland Ziekenhuis, Leiderdorp: C. van Rees, C.J.H.J. Kirchhof

Rode Kruis Ziekenhuis, Beverwijk: L. Konst

Slingeland Ziekenhuis, Doetinchem: H. Drost

Slotervaartziekenhuis, Amsterdam: R.A.M. van Liebergen

St. Anna Ziekenhuis, Geldrop: P.E. Polak

St. Antonius Ziekenhuis, Nieuwegein: H.W.M. Plokker

St. Lucas Andreas Ziekenhuis, Amsterdam: J. Schroeder-Tanka

Tergooi ziekenhuis, Hilversum: P. de Milliano

Twee Steden Ziekenhuis, Tilburg: H. van Kesteren

IJsselland Ziekenhuis, Capelle a/d IJssel: B.J. van den Berg

Zaans Medisch Centrum, Zaandam: P.N.A. Bronzwaer

Ziekenhuis Gelderse Vallei, Ede: T.T. van Loenhout

*Laboratories*

[National Institute of Public Health and the Environment](http://www.onderzoekinformatie.nl/en/oi/nod/organisatie/ORG1236213/toon) (RIVM), Bilthoven: E.H.J.M. Jansen

Stichting Huisartsenlaboratorium Oost (SHO), Velp: W. Grootaarts, D. van Rumpt

Wageningen University, Division of Human Nutrition, Wageningen: P.J.M. Hulshof, H.M. van der Struijs-van de Putte, P. Versloot, R. Hovenier.

*Dietetics*

Wageningen University, Division of Human Nutrition, Wageningen: J.H.M. de Vries, E. Siebelink

*Trial margarines*

Unilever R&D, Vlaardingen: O.E. Rosier, J.L. Zevenbergen

All of the abovementioned centers are located in the Netherlands
